# Cerebral cortical thickness and cognitive decline in Parkinson’s disease

**DOI:** 10.1093/texcom/tgac044

**Published:** 2023-01-14

**Authors:** Colleen Pletcher, Kevin Dabbs, Amy Barzgari, Vincent Pozorski, Maureen Haebig, Sasha Wey, Stephanie Krislov, Frances Theisen, Ozioma Okonkwo, Paul Cary, Jennifer Oh, Chuck Illingworth, Michael Wakely, Lena Law, Catherine L Gallagher

**Affiliations:** William S. Middleton Memorial Veterans Hospital, Madison, WI, United States; Department of Neurology, University of Wisconsin School of Medicine and Public Health, Madison, WI, United States; Department of Neurology, University of Wisconsin School of Medicine and Public Health, Madison, WI, United States; William S. Middleton Memorial Veterans Hospital, Madison, WI, United States; Department of Neurology, University of Wisconsin School of Medicine and Public Health, Madison, WI, United States; William S. Middleton Memorial Veterans Hospital, Madison, WI, United States; Department of Neurology, University of Wisconsin School of Medicine and Public Health, Madison, WI, United States; William S. Middleton Memorial Veterans Hospital, Madison, WI, United States; Department of Neurology, University of Wisconsin School of Medicine and Public Health, Madison, WI, United States; Medical College of Wisconsin, Milwaukee, WI, United States; Institute for Clinical and Translational Research, Madison, WI, United States; Cox Medical Centers, Department of Surgery, Springfield, MO, United States; William S. Middleton Memorial Veterans Hospital, Madison, WI, United States; Wisconsin Alzheimer’s Disease Research Center, Madison, WI, United States; Wisconsin Alzheimer’s Disease Research Center, Madison, WI, United States; William S. Middleton Memorial Veterans Hospital, Madison, WI, United States; Wisconsin Alzheimer’s Disease Research Center, Madison, WI, United States; William S. Middleton Memorial Veterans Hospital, Madison, WI, United States; Wisconsin Alzheimer’s Disease Research Center, Madison, WI, United States; Wisconsin Alzheimer’s Disease Research Center, Madison, WI, United States; Wisconsin Alzheimer’s Disease Research Center, Madison, WI, United States; William S. Middleton Memorial Veterans Hospital, Madison, WI, United States; Department of Neurology, University of Wisconsin School of Medicine and Public Health, Madison, WI, United States; Wisconsin Alzheimer’s Disease Research Center, Madison, WI, United States

**Keywords:** aged, cerebral cortex/pathology, cognitive dysfunction/etiology, magnetic resonance imaging, humans

## Abstract

In Parkinson’s disease (PD), reduced cerebral cortical thickness may reflect network-based degeneration. This study performed cognitive assessment and brain MRI in 30 PD participants and 41 controls at baseline and 18 months later. We hypothesized that cerebral cortical thickness and volume, as well as change in these metrics, would differ between PD participants who remained cognitively stable and those who experienced cognitive decline. Dividing the participant sample into PD-stable, PD-decline, and control-stable groups, we compared mean cortical thickness and volume within segments that comprise the prefrontal cognitive-control, memory, dorsal spatial, and ventral object-based networks at baseline. We then compared the rate of change in cortical thickness and volume between the same groups using a vertex-wise approach. We found that the PD-decline group had lower cortical thickness within all 4 cognitive networks in comparison with controls, as well as lower cortical thickness within the prefrontal and medial temporal networks in comparison with the PD-stable group. The PD-decline group also experienced a greater rate of volume loss in the lateral temporal cortices in comparison with the control group. This study suggests that lower thickness and volume in prefrontal, medial, and lateral temporal regions may portend cognitive decline in PD.

## Introduction

Parkinson’s disease (PD) is a progressive neurodegenerative disorder characterized by motor symptoms of tremor, bradykinesia, rigidity, and postural instability, as well as non-motor symptoms that frequently include cognitive decline, placing strain on caregivers and the healthcare system (Goldman et al. 2018). Cortical thickness, measured via structural brain magnetic resonance imaging (MRI), has been found to be lower in PD patients (Lyoo et al. 2010; Pereira et al. 2014; Koshimori et al. 2015; Gerrits et al. 2016; Luo et al. 2016; Yau et al. 2018; Zhang et al. 2018), and to be a distinguishing feature of cognitive status and disease stage (Song et al. 2011; Biundo et al. 2013; Pagonabarraga et al. 2013; Zarei et al. 2013; Segura et al. 2014; Danti et al. 2015; Herb et al. 2016; Gasca-Salas et al. 2019; Laansma et al. 2021). Although many studies have demonstrated reduced cortical thickness in PD with dementia or mild cognitive impairment (MCI), few have parsed domain-specific relationships (Filoteo et al. 2014). Despite recent efforts to assess regional cortical thickness longitudinally as a correlate of cognitive change, differences between cognitively impaired and unimpaired PD patients have been more difficult to discern than those between PD and control, and studies disagree regarding which regions are most affected (Ibarretxe-Bilbao et al. 2012; Compta et al. 2013; Hanganu et al. 2014; Mak et al. 2015; Caspell-Garcia et al. 2017; Garcia-Diaz et al. 2018; Filippi et al. 2020).

The first aim of the present study was to evaluate the degree to which cognitive course (stable versus decline) differentiated mean cerebral cortical thickness within the cortical portions of pre-specified functional networks at baseline. The human brain is connected through a complex series of cortical–subcortical networks of which anatomically connected regions that show synchronous blood-oxygen-dependent, slow signal fluctuations at rest (Seeley et al. 2009; Yeo et al. 2011). The network degeneration hypothesis predicts that degenerative changes should occur selectively within systems that recapitulate healthy functional network architecture. Indeed, atrophy patterns do follow these intrinsic connectivity networks (ICNs) in a variety of syndromes (Seeley et al. 2009), as well as in PD (Zeighami et al. 2015; Yau et al. 2018). For this study, we hypothesized that network-based degeneration would cause early, selective reductions in cortical thickness within regions that serve specific cognitive roles known to be impaired in PD. Thus, we computed the average thickness and volume of FreeSurfer-derived cortical segments that participate in 4 cognitive networks (prefrontal cognitive control, medial temporal memory, dorsal spatial-based, and ventral object-based systems) based on a previous cross-sectional investigation that related domain-specific cognitive functions to regional volumes in PD (Filoteo et al. 2014). Previous studies of PD have reported deficits in cognitive functions supported by each of these systems, including visuospatial processing and attention, cognitive flexibility, working memory, motor planning, and decision-making (Kehagia et al. 2010; Kravitz et al. 2013; Ptak et al. 2017). We hypothesized that PD patients who experienced cognitive decline over the study would have thinner cortex in one or more of these networks at baseline. Posterior cingulate and precentral gyral thicknesses were also investigated based on thickness differences shown in previous Parkinson’s studies (Pereira et al. 2012; Pagonabarraga et al. 2013; Mak et al. 2015; Koshimori et al. 2015; Uribe et al. 2016; Zhang et al. 2018).

The second aim was to evaluate longitudinal rates of cortical volume and thickness change in relation to cognitive course. Cross-sectional studies of PD have shown a greater degree of cortical thinning at more advanced stages of disease and with more significant cognitive, motor, and non-motor impairment (Fereshtehnejad et al. 2017; Laansma et al. 2021). Other studies suggest that the greatest detectable rate of cortical thinning occurs in early-stage disease, during the transition from normal cognition to MCI, in contrast to MCI to dementia (Filippi et al. 2020). To address this aim, we used a vertex-wise approach to improve sensitivity, given the short time span (1.5 years) between baseline and follow-up visits. We hypothesized that PD patients who experienced cognitive decline would have a greater reduction in cortical thickness and volume over the 18 months of the study in comparison to stable participants.

## Materials and methods

### Participants

Eighty-five participants were recruited through local neurology clinics and the Wisconsin Alzheimer’s Disease Research Center as part of the VA-sponsored Longitudinal MRI in Parkinson’s Disease (LMPD) study. Participants completed 2 visits 18 months apart, providing written informed consent at the first study visit prior to research procedures. Enrollees were free of significant cardiovascular and cerebrovascular disease, known major psychiatric or neurological disease other than PD, and were required to have a Mini Mental State Examination (MMSE; Folstein et al. 1975) score of 27/30 or higher at baseline. To exclude genetic cases, PD participants were excluded if they experienced initial motor symptoms earlier than 45 years of age, or if they had more than one first-degree relative with PD. Both visits consisted of a neuropsychological assessment, Unified Parkinson’s Disease Rating Scale (UPDRS; Fahn and Elton 1987) scoring by a movement disorders neurologist (C.G.), and brain MRI. PD participants were off anti-Parkinson medications for 12–18 h prior to each visit. Of the 85 enrollees, 71 (41 controls and 30 PD) completed both the baseline and 18-month follow-up visits. After image processing, 66 enrollees had Freesurfer outputs of sufficient quality for the cross-sectional analysis, and 55 for the longitudinal analysis. The study was approved by the University of Wisconsin Health Sciences Institutional Review Board (IRB) and by the William S. Middleton VA R&D committee.

### Neuropsychological assessment

A cognitive battery designed to evaluate performance within the domains of literacy, executive function, memory, and language, was administered at both baseline and follow-up visits. Although visuospatial deficits have been described in PD, this domain was not specifically evaluated in the present study. Tests administered included the Wide Range Achievement Test—Word reading subset score Fourth Edition (WRAT-IV) reading test (Wilkinson and Robertson 2006), category (Goodglass and Kaplan 1972; Rosen 1980) and phonemic fluency tests (C–F–L form; Benton and Hamsher 1976), Trail Making Tests (TMT) A and B (Reitan 1992), Wisconsin Card Sort Test—64 (WCST-64; Grant and Berg 1948; Heaton et al. 1993), Hopkins Verbal Learning Test (HVLT; Brandt 1991; Benedict et al. 1998), and Boston Naming Test (BNT; Kaplan et al. 1983). The WRAT-IV was used as an index of the overall quality of educational attainment in lieu of formal years of education (Manly et al. 2002; Rohit et al. 2007; Sayegh et al. 2014). The TMT test is unlike other tests included in this battery, since a lower score indicates a faster time and thus better cognitive performance. The difference of sign for TMT is reflected in the method for generating composite scores.

To improve the stability of cognitive measures, individual task scores or sub-scores were combined to create composite measures within the domains of executive functioning, memory, and language. To accomplish this, individual raw task scores from both baseline and follow-up visits were converted into *z*-scores based on the control group mean and standard deviation (SD) at baseline. Then, individual visit-specific task *z*-scores were combined as follows: “Executive function composite” = [(Category Fluency + WCST-64 categories completed—{TMT Part B time—TMT Part A time})/3]; “Memory composite” = [(HVLT delayed recall + recognition discrimination index)/2]; “Language composite” = [(BNT + Phonemic Fluency)/2]. Change scores within these 3 cognitive domains were generated by subtracting the baseline composite *z*-score from the respective follow-up score. “Decline,” defined as reduction of ≥1 SD in 2 or more domains between baseline and follow-up visits, was used to divide the cohort into 4 groups: Control-stable; Control-decline; PD-stable; and PD-decline. Based on this threshold, 36 control and 20 PD participants remained cognitively stable over the 18-month study, whereas 10 PD subjects and 5 controls declined. As this study was focused on predictors of cognitive decline in PD, the 5 control-decliners were removed from further analysis.

### Brain magnetic resonance imaging acquisition

A GE 750 Discovery 3T MRI system (General Electric Healthcare, Waukesha, WI) with an 8-channel phased array head coil was used for the acquisition of brain MRI data. A high-resolution 3-dimensional brain volumetric (BRAVO) T1-weighted inversion prepared sequence of inversion time (TI) = 450 ms, repetition time (TR) = 8.2 ms, echo time (TE) = 3.2 ms, flip angle = 12°, acquisition matrix = 256 × 256, field of view (FOV) = 256 mm, and slice thickness = 1.0 mm collected in the axial plane was acquired for cortical parcellation.

### Systems-based analysis

For the systems-based cross-sectional analysis, T1-weighted MRI volumes were automatically segmented using the FreeSurfer image analysis suite version 5.1.0 (Fischl et al. 1999, 2002; Ségonne et al. 2004; Jovicich et al. 2006; Reuter et al. 2010). T1-weighted volumes were skull stripped, intensity normalized, and transformed to Talairach space, after which FreeSurfer produced surface meshes along the pial/CSF and gray/white matter boundaries (Dale et al. 1999; Fischl and Dale 2000). These surfaces were visually inspected and manually edited by 2 researchers (A.W. and F.T.) who were blind to subject’s diagnosis, and who had an inter-rater reliability Spearman’s correlation of 0.976 based on thickness measurements of 5 subjects. Six subjects were excluded due to quality of the FreeSurfer outputs, leaving a total of 66 for the cross-sectional analysis. After these manual edits, images were reprocessed and re-inspected prior to the acquisition of final regional thicknesses, which FreeSurfer computes by averaging the distance along a normal vector from the white matter surface to the pial surface at each vertex within the respective region of interest (ROI). The cortex was parcellated according to the Desikan–Killiany cortical atlas to produce 34 cortical ROIs in each hemisphere (68 total ROIs; Desikan et al. 2006). Based on the network hypothesis of neurodegeneration (Seeley et al. 2009), we expected that volume and thickness change would affect functionally related networks of anatomic regions. So, we averaged cortical thickness and volume across bilateral components of the prefrontal cognitive control (rostral and caudal anterior cingulate, rostral and caudal middle frontal, superior frontal, pars triangularis, pars opercularis, and pars orbitalis), medial temporal memory (temporal pole, entorhinal, and parahippocampal), dorsal spatial-based (superior and inferior parietal), and ventral object-based (superior, middle, and inferior temporal, fusiform, lingual, and lateral occipital) systems based on Filoteo et al. (2014). The precentral gyrus and posterior cingulate cortex were also chosen for analysis due to thickness or volumetric differences between PD and controls described in previous literature (Pereira et al. 2012; Pagonabarraga et al. 2013; Zarei et al. 2013; Koshimori et al. 2015; Mak et al. 2015; Uribe et al. 2016; Zhang et al. 2018; Gasca-Salas et al. 2019).

### Longitudinal image processing

Nine control-stable participants and 2 PD-decline participants included in the cross-sectional analysis were excluded from the longitudinal analysis due to severe banding (5 participants) or motion (6 participants) on their second timepoint T1 image, leaving 27 control-stable, 20 PD-stable, and 8 PD-decline participants (Table 1). Longitudinal processing was accomplished by resampling each individual’s T1 volumes to an unbiased template, or “base,” representing the average of these 2 timepoints in FreeSurfer Version 6 (Reuter and Fischl 2011). Each base image was inspected and manually edited as needed by 2 raters (A.B. and C.P.) who were blind to participant diagnosis. Then, longitudinal outputs were created by mapping individual timepoint images to the base template and running this created image through the FreeSurfer longitudinal processing stream, thus creating “longitudinal” outputs for each visit (Reuter et al. 2012), which were visually inspected.

**Table 1:** Baseline characteristics of participants included in cross-sectional and longitudinal analyses

Baseline cross-sectional	Control-stable (*n* = 36)	PD-Stable (*n* = 20)	PD-decline (*n* = 10)	*P*-value
Age (years)	65.9 (7.5)	62.6 (8.8)	73.6 (6.8)	0.003^a^^,^^b^^,^^c^
Time between visits (years)	1.48 (0.06)	1.51 (0.09)	1.51 (0.08)	0.229
Sex (Male/Female)	27/9	16/4	8/2	0.921^†^
WRAT-IV	62.4 (3.8)	62.0 (5.5)	61.4 (4.9)	0.831
MMSE	29.6 (0.6)	29.0 (0.99)	28.7 (0.8)	0.002^a^^,^^b^
Age at diagnosis (years)	N/A	59.2 (8.7)	69.1 (6.2)	0.003^c^
Disease duration (years)	N/A	3.4 (3.2)	4.6 (3.4)	0.385
UPDRS motor sub-score	1.4 (1.6)	20.7 (12.1)	20.4 (8.4)	< 0.001^a^^,^^b^
UPDRS total score	2.7 (3.0)	35.6 (15.3)	33.9 (12.1)	< 0.001^a^^,^^b^
H&Y staging	N/A	1.65 (.65)	1.6 (.57)	1.00
				
**Longitudinal**	**(*n* = 27)**	**(*n* = 20)**	**(*n* = 8)**	
Age (years)	65.3 (7.0)	62.6 (8.8)	72.6 (7.2)	0.013^c^
Time between visits (years)	1.48 (0.07)	1.51 (0.09)	1.52 (0.09)	0.215
Sex (Male/Female)	22/5	16/4	6/2	0.904^†^
WRAT-IV	61.7 (3.6)	62.0 (5.5)	62.9 (4.2)	0.802
MMSE	29.6 (0.63)	29.0 (0.99)	28.8 (.89)	0.011^b^
Age at diagnosis (years)	N/A	59.2 (8.7)	68.6 (6.7)	<0.001^c^
Disease duration (years)	N/A	3.4 (3.2)	3.9 (3.5)	0.400
UPDRS motor sub-score	1.2 (1.3)	20.7 (12.1)	20.0 (8.9)	<0.001^a^^,^^b^
UPDRS total score	2.4 (2.7)	35.6 (15.3)	33.0 (12.6)	<0.001^a^^,^^b^
H&Y staging	N/A	1.65 (65)	1.4 (.50)	0.740

Mean (standard deviation) are shown except in column 4, which shows the ANOVA *P*-value, with significant between-group differences indicated as follows,

^a^PD-stable versus control-stable.

^b^PD-decline versus control-stable.

^c^PD-decline versus PD-stable.

^†^Fisher’s Exact Probability test was used to compare sex distribution.

Abbreviations: MMSE, Mini-Mental State Examination; N/A, not applicable; PD, Parkinson’s disease; WRAT-IV, Wide Range Achievement Test—Fourth Edition word reading sub scale; UPDRS, Unified Parkinson’s Disease Rating Scale.

### Cross-sectional statistics

Statistical analyses were conducted in International Business Machines’ Statistical Program for the Social Sciences (IBM SPSS, Version 22.0; Chicago). Demographic and disease characteristics were compared between groups (control-stable, PD-stable and PD-decline) using 1-way analysis of variance (ANOVA) for continuous variables and Fisher’s exact test for categorical variables such as sex distribution. Composite neuropsychological scores at baseline and rates of change in these scores were compared across groups using 1-way analyses of covariance (ANCOVA), controlling for age, sex, educational attainment (WRAT-IV), and time between visits. Post-hoc pairwise tests were corrected for multiple comparisons at *P* < 0.05 using Bonferroni method (*P* < 0.05/3), and false discovery rate correction across the 6 ANCOVAs was conducted using Benjamini–Hochberg procedure (α = 0.05). Baseline mean cortical thicknesses and volume within the 4 major cognitive networks, as well as precentral gyrus and posterior cingulate gyrus, were compared between control-stable, PD-stable, and PD-decline groups in 6 ANCOVAs, controlling for age, sex, and educational attainment. The distribution of regional thicknesses or volume within each group were evaluated using the Shapiro–Wilk test for normality and were found to meet criteria for normality, and residuals were checked for homoscedasticity. Within each ANCOVA, pairwise post-hoc tests were corrected for multiple comparisons using Bonferroni method at alpha = 0.05, (corrected *P*-value < 0.05/3). Multiple tests correction for the 6 ANCOVAS was performed using the Benjamini–Hochberg procedure with a false discovery rate (FDR) of 0.05. Two additional post-hoc analyses were conducted: (i) Because the subject groups were not ideally matched for age, the PD-decline group was compared with a subgroup of 10 age-matched controls using a paired samples *t*-test to confirm that age was not confounding the results; (ii) 2 PD subjects had 2 cognitive domain scores > 1.5 SD below mean at baseline, meeting the criteria set by Litvan et al. (2012) for mild cognitive impairment. Therefore, between-group ANCOVAs and correction for multiple comparisons were repeated after the removal of these 2 subjects to confirm that inclusion of these subjects did not change the results.

### Longitudinal statistics

Vertex-wise cortical thickness and volume maps of symmetrized percent change (rate of change between the 2 timepoints divided by the mean thickness or volume at each vertex multiplied by 100) generated using FreeSurfer (version 6)’s longitudinal processing stream Query, Design, Estimate, Contrast (QDEC) processing, smoothed at 10-mm full width half max (FWHM), were compared between control-stable, PD-stable, and PD-decline groups using generalized linear models while controlling for age in the cortical thickness analyses, and age and total intracranial volume in the volume analyses. Monte Carlo simulation was used to test for statistically significant clusters, with cluster-wise *P*-values set at *P* < 0.05.

## Results

### Demographic and group characteristics

Results of 1-way ANOVAs comparing demographic and disease characteristics at baseline between control-stable (*n* = 36), PD-stable (*n* = 20), and PD-decline (*n* = 10) groups are summarized in Table 2. Results of 1-way ANOVAs comparing demographic and disease characteristics at baseline of the longitudinal groups between control-stable (*n* = 27), PD-stable (*n* = 20), and PD-decline (*n* = 8) groups are summarized in Table 3. Quality of education (WRAT-IV), sex, and time between visits did not differ significantly between-groups. However, the PD-decline group was older (73.6 ± 6.8 years) than both the PD-stable (mean 62.6 ± 8.8 years) and control-stable (mean 65.9 ± 7.5 years) groups. Although all MMSE scores were within normal limits of ≥27 as a criterion for enrollment, mean scores were significantly lower in the PD-stable and PD-decline groups compared with the control-stable group in the cross-sectional analysis, and significantly lower in the PD-decline group compared with the control-stable group for the longitudinal analysis. Disease duration did not differ between PD subgroups. Commensurate with the PD-decline group being older, mean age at diagnosis was 59.2 (± 8.7) for PD-stable and 69.1 (±6.2) for PD-decline; however, the disease duration did not differ significantly between the PD groups. All but 2 PD participants were taking anti-Parkinson medications (dopamine agonists, levodopa, amantadine, and/or monoamine oxidase inhibitors) at the time of study enrollment. As expected, PD participants had significantly higher scores on both the total and motor sub-section of the UPDRS than controls; however, baseline UPDRS scores did not differ between PD-stable and PD-decline groups.

**Table 2:** Baseline composite *z*-scores and longitudinal change for neuropsychological domains

	Control-stable (*n* = 36)	PD-stable (*n* = 20)	PD-decline (*n* = 10)	*P*-value (*F*)
Executive function composite *z-score*_Baseline_	0.07 (0.12)	−0.48 (0.16)	−0.75 (0.24)	0.001 (7.93)^a^^,^^b^
Executive function composite *z-score*_∆_	−0.02 (0.10)	−0.02 (0.14)	−0.95 (0.20)	0.000 (8.78)^b^^,^^c^
Memory composite *z-score*_Baseline_	0.02 (0.20)	−0.73 (0.27)	−0.91 (0.40)	0.022 (4.09)
Memory composite *z-score*_∆_	0.35 (0.13)	0.09 (0.17)	−0.59 (0.25)	0.006 (5.54)^b^
Language composite *z-score*_Baseline_	0.06 (0.13)	−0.03 (0.18)	−0.30 (0.26)	0.471 (0.76)
Language composite *z-score*_∆_	0.21 (0.13)	0.23 (0.17)	−0.11 (0.25)	0.499 (0.70)

Composite *Z*-score mean (standard deviation) at baseline and change (∆) over the 18-month interval. *P*-values (column 4) were derived via ANCOVA. The ANCOVAs for the change scores controlled for age, sex, WRAT-IV, and time between visits. Significant between-group differences following multiple comparisons correction are indicated as follows.

^a^PD-stable and control-stable.

^b^PD-decline and control-stable.

^c^PD-decline and PD-stable.

### Neuropsychological test performance

Baseline neuropsychological composite scores and mean change in these scores between baseline and 18-month follow-up are summarized in Table 2. At baseline, executive composites were lower in both the PD-decline and PD-stable groups in comparison with the control-stable group but did not differ significantly between the PD groups. Two PD subjects scored >1.5 SD below the control mean in 2 cognitive domains at baseline, thus meeting criteria for MCI (Litvan et al. 2012). As would be expected based on our scheme for classifying subjects as decline versus stable, the PD-decline group showed a greater change in executive function and memory over the study interval than the PD-stable and control-stable groups. The improvement of the control-stable and PD-stable groups on the memory and language composites could reflect practice effects.

### Baseline cortical thickness and cognitive decline

**Fig. 1:**
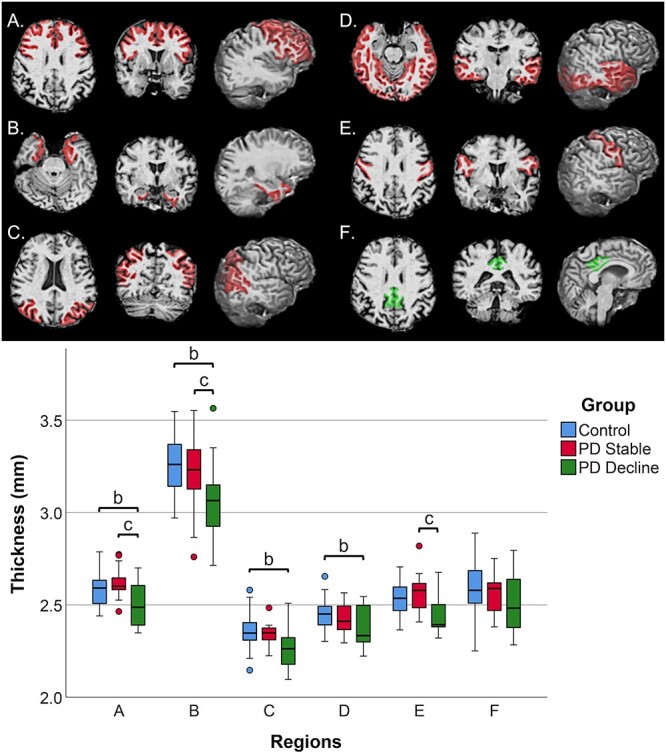
Regional cortical thickness comparisons between PD subgroups and controls *Upper panel:* Axial, coronal, and sagittal views of regions within the A) prefrontal cognitive-control system, B) medial temporal memory system, C) dorsal spatial-based system, D) ventral object-based system, E) precentral gyrus, and F) posterior cingulate cortex. Regions in red represent systems with significant differences in mean thickness between control-stable, PD-stable, and PD-decline observed via 1-way ANCOVA. Regions in green are nonsignificant. *Lower panel:* Boxplots display mean thickness for each region (A–F) between control-stable, PD-stable, and PD-decline, with significant between group differences indicated by “b” and “c.”

The results of one-way ANCOVA analyses comparing baseline cortical thickness between control-stable, PD-stable, and PD-decline groups are summarized in Table 3 and depicted in Fig. 1. The PD-decline group had lower mean cortical thickness than the control group for every system analyzed with the exception of the posterior cingulate and precentral gyri. In addition, the PD-decline group had lower mean cortical thickness than the PD-stable group within the prefrontal cognitive-control system, medial temporal memory system, and precentral gyrus. Mean cortical thickness of the regions evaluated did not differ significantly between PD-stable and control participants. Repeating this analysis after excluding the 2 PD participants who met criteria for MCI at baseline replicated these results. Because the PD-decline group was significantly older than the comparison groups, a second subgroup analysis was conducted by matching 10 PD-decline subjects (mean age = 73.65 ± SD 6.84 years) with 10 control-stable subjects (mean age = 73.60 ± SD 7.18 years). Paired *t*-tests comparing cortical thickness between these groups showed that the PD-decline group had lower cortical thickness (*P* < 0.05) within the temporal memory and dorsal spatial-based systems, and a trend towards lower thickness in the prefrontal cognitive control and ventral object-based systems, as well as precentral gyrus (*P* < 0.10 for all). We did not find significant differences in systems-defined volume between the participant groups.

**Table 3:** Baseline cortical thickness comparisons between PD subgroups and controls

Mean cortical thickness (mm)	Control-stable (*n* = 36)	PD-stable (*n* = 20)	PD-decline (*n* = 10)	*F* (df 2, 59)	*P*-value
Prefrontal cognitive-control	2.58 (0.08)	2.62 (0.08)	2.50 (0.12)	6.583	0.003^b^^,^^c^
Medial temporal memory system	3.26 (0.15)	3.22 (0.20)	3.07 (0.26)	5.851	0.004^b^^,^^c^
Ventral object-based system	2.45 (0.08)	2.42 (0.08)	2.37 (0.11)	3.873	0.032^b^
Dorsal spatial-based system	2.36 (0.09)	2.34 (0.06)	2.27 (0.12)	3.925	0.050^b^
Precentral gyrus	2.54 (0.09)	2.56 (0.10)	2.44 (0.10)	3.678	0.041^c^
Posterior cingulate	2.60 (0.12)	2.56 (0.10)	2.51 (0.18)	1.697	0.117

Mean (standard deviations) cortical thickness (mm) for each system/ROI was compared between control-stable, PD-stable, and PD-decline groups using ANCOVA with covariates of age, sex, and educational achievement (WRAT-IV). Post-hoc pairwise tests were corrected within each ANCOVA using Bonferroni method (*P* < 0.05/3), after which p-values of the 6 ANCOVAs were FDR-corrected using Benjamini–Hochberg procedure (α = 0.05). Significant between-group differences following these multiple comparisons corrections are indicated as follows.

^b^PD-decline versus control-stable.

^c^PD-decline versus PD-stable.

*Abbreviations:* ANCOVA, analysis of covariance; PD, Parkinson’s disease; df, degrees of freedom.

### Longitudinal rates of volume and thickness change

**Fig. 2:**
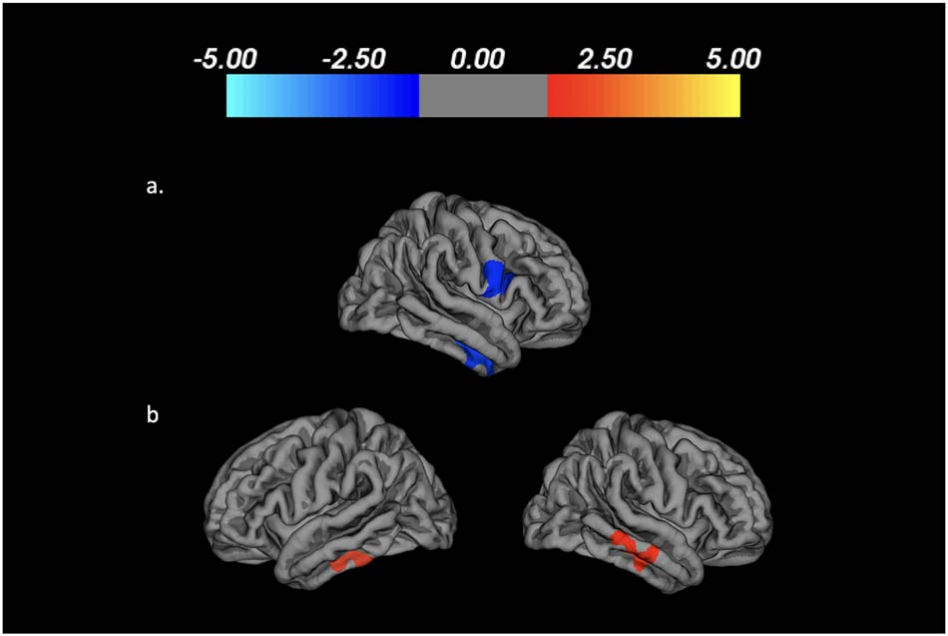
Longitudinal vertex-wise results: The color bar represents *T*-values for significant clusters where a) symmetrized percent change in cortical thickness was greater in Control-stable than PD-stable participants, reflecting a relative increase in cortical thickness in controls; b) symmetrized percent decrease in cortical volume was greater in PD-decline versus Control-stable participants.

The results of vertex-wise comparison of rates of change in cerebral cortical thickness and volume between participant groups over 18 months are shown in Table 4 and Fig. 2. Over the 1.5-year study, a greater increase in cortical thickness was detected in the control-stable group than the PD-stable group in clusters encompassing the right middle temporal gyrus and left pars opercularis. The PD-decline group showed greater reductions in volume of the left inferior and right middle temporal gyri in comparison with the control group.

**Table 4:** Longitudinal vertex-wise results

	Control > PD-stable	PD-Decline > Control
Cortical thickness increase	R Middle temporal (748.71); L pars opercularis (691.42)	
Cortical volume decrease		L Inferior temporal (593.22); R middle temporal (593.22)

Percent change in cortical thickness (mm) or volume (mm^3^) between the 2 visits was compared between control, PD-stable, and PD-decline groups using general linear models with age as a covariate. The location and size of significant clusters (mm^2^), cluster-corrected at 0.05, are shown in the table.

***Abbreviations:*** L = left hemisphere, R = right hemisphere.

## Discussion

Cortical thinning in networks functionally connected with the substantia nigra and striatum has been described in PD, and hypothesized to be due to propagation of misfolded proteins from a subcortical disease reservoir (Zeighami et al. 2015; Yau et al. 2018). Most studies have described a greater extent of cortical thinning in PD-MCI and PD dementia than PD with normal cognition, and more extensive subcortical and cortical volume loss in the “malignant” PD phenotype as defined by motor and cognitive impairment, dysautonomia, and dependency for activities of daily living (Fereshtehnejad et al. 2017; Laansma et al. 2021). However, reports of specific relationships between regional cortical volume/thickness and domain-specific cognitive function are limited (Filoteo et al. 2014), in part because many studies have not performed detailed cognitive testing. In this study, we attempted to parse the relationship between domain-based measures of cognitive decline for memory, executive function, and language with mean cortical thickness in networks of regions combined a priori based on their known cognitive roles. However, we found that within the PD-decline (i.e. “malignant”) subgroup, cortical thinning was fairly diffuse, involving multiple anatomic “systems,” at baseline, in comparison with the control group. Given that PD-decline participants also had lower cognitive scores at baseline, we hypothesize that their accelerated cortical thinning had commenced prior to study enrollment, and possibly even prior to motor symptom onset. In fact, thinning of frontal, parietal, and occipital cortices has been described in idiopathic Rapid Eye Movement Sleep Behavior Disorder patients who convert to Lewy body disease (Pereira et al. 2019).

When compared with the PD-stable subgroup, the PD-decline subgroup had lower mean cortical thickness in the prefrontal cognitive control and medial temporal memory systems, as well as the precentral gyrus. These systems support cognitive functions that are vulnerable to change in PD, especially attention/executive function, and memory encoding (Muslimović et al. 2005). We did not see thinning in the posterior cingulate cortex, as can be seen in Alzheimer disease and has been described in some previous studies of PD (Leech et al. 2013). Although we aspire to develop biomarkers to predict cognitive decline in PD, the overlap between control and PD thicknesses in these regions will preclude their use as predictive measures for individual patients.

Cross-sectional studies that have compared PD and control groups have shown PD patients to have lower cortical thickness in prefrontal, temporal, parietal, and occipital regions (Lyoo et al. 2010; Pereira et al. 2012; Biundo et al. 2013; Pagonabarraga et al. 2013; Zarei et al. 2013; Garcia-Diaz et al. 2014; Pereira et al. 2014; Danti et al. 2015; Gerrits et al. 2016; Uribe et al. 2016; Zhang et al. 2018; Gasca-Salas et al. 2019; Filippi et al. 2020) as well as precentral gyrus, precuneus, and insula (Biundo et al. 2013; Pagonabarraga et al. 2013; Hanganu et al. 2014; Danti et al. 2015; Mak et al. 2015; Zhang et al. 2018; Gasca-Salas et al. 2019; Filippi et al. 2020). Lower cortical thickness in PD-MCI compared with PD-normal cognition have been shown in medial temporal (Biundo et al. 2013), superior frontal (Biundo et al. 2013; Pagonabarraga et al. 2013; Danti et al. 2015), inferior parietal and supramarginal gyri (Biundo et al. 2013; Segura et al. 2014), precuneus (Pereira et al. 2014; Segura et al. 2014), and precentral gyrus (Pagonabarraga et al. 2013). A recent large multicenter study showed cortical thinning in 38 of 68 standard Freesurfer parcels in PD, with more extensive and severe thinning at more advanced stages of disease (Laansma, 2021). For smaller studies, achieving age-matched groups has been difficult, since PD-MCI and PD dementia participants tend to be older than unimpaired groups (Biundo et al. 2013; Pagonabarraga et al. 2013; Pereira et al. 2014; Segura et al. 2014; Mak et al. 2015), and advanced age and older age of disease onset are known risk factors for PD dementia (Emre et al. 2007). Studies of PD dementia in which samples were relatively age-matched have not shown significant regional cortical thinning but have shown lower global cortical thickness in comparison with controls (Colloby et al. 2020; Filippi et al. 2020). Hippocampal and medial temporal lobe atrophy have also been correlated with cognitive impairment in some studies (Weintraub et al. 2011).

Longitudinal studies using FreeSurfer have shown a 0.3–0.7% annual decline in cortical thickness within most cortical regions in healthy adults, with variation in rates between regions (Weintraub et al. 2011). Modeling of the effects of aging on cortical thickness based on cross sectional data from Parkinson’s Progression Markers Initiative (PPMI) and Alzheimer’s Disease Neuroimaging Initiative (ADNI) suggest that patients with PD may have a different from normal trajectory of cortical thinning, with accelerated atrophy in frontal and precuneus regions (Claassen et al. 2016). PD-MCI patients have been shown to have accelerated rates of thinning in prefrontal and anterior cingulate (Caspell-Garcia et al. 2017; Hanganu et al. 2014; Ibarretxe-Bilbao et al. 2012; Mak et al. 2015), inferior parietal (Garcia-Diaz et al. 2018), lateral temporal (Hanganu et al. 2014; Mak et al. 2015), lateral occipital (Garcia-Diaz et al. 2018), and insular cortex (Hanganu et al. 2014), as well as temporal pole (Hanganu et al. 2014). Longitudinal comparisons of the rate of cortical thinning between PD patients with normal cognition, those who transition from normal to MCI, those with stable MCI, and those who transition from MCI to dementia, suggests that cortical thinning exceeds the control rate in multiple and diffuse regions at earlier stages of disease (PD-normal cognition and PD-normal cognition to MCI) (Filippi et al. 2020). These results are commensurate with those of our study, both of which suggest that in PD patients with a “malignant” phenotype who are destined to experience cognitive decline, cortical thinning occurs prior to disease diagnosis and clinically significant cognitive decline.

The longitudinal vertex-wise analysis of our sample showed greater rates of volume loss involving right middle and left inferior temporal gyri in PD-decline compared with control participants, with no detectable differences between PD-stable and control participants. Recent studies suggest that volumetric differences in PD affect predominantly subcortical structures (Filippi et al. 2020; Laansma et al. 2021), which were not investigated in this study. Nonetheless, many studies have shown lower cortical thickness or increased rates of thinning of the middle and inferior temporal cortices, especially in PD-MCI (Lyoo et al. 2010; Ibarretxe-Bilbao et al. 2012; Pereira et al. 2012; Pagonabarraga et al. 2013; Hanganu et al. 2014; Segura et al. 2014; Danti et al. 2015; Mak et al. 2015; Gasca-Sales 2017; Yau et al. 2018; Zhang et al. 2018; Laansma et al. 2021). Our volumetric findings likely reflect a similar phenomenon. The inferior temporal and fusiform cortex are part of the ventral visual (“what”) pathway and with occipital cortices show hypometabolism in Lewy body dementia (Caminiti et al. 2019). Injection of the nonhuman primate lateral temporal cortex with herpesvirus traces connections to substantia nigra pars reticulata via the thalamus, providing a potential mechanism for transfer of “prions” from a “reservoir” of neurodegeneration (Middleton and Strick 1996).

## Conclusion

The present study was limited by a relatively small sample size, especially for the longitudinal analyses, by lack of perfect age-matching between the PD-stable and PD-decline groups, and by lack of specific visuospatial assessment tools. Nonetheless, the statistical analyses were controlled for age, and the age-matched subgroup analysis recapitulated results derived from the entire sample. The study benefited from more detailed cognitive characterization of the participants than has been available to many prior studies, as well as MR images acquired at the same site and on the same scanner between the baseline and follow-up visits. Taken together, the present study and previous studies suggest that Parkinson’s disease affects the cerebral cortex in early and even preclinical stages of disease. Cortical thickness within the prefrontal cognitive control and medial temporal memory systems may differentiate PD patients who will remain cognitively stable from those who will experience cognitive decline. Cognitive progression in PD may be associated with volume loss in the lateral temporal cortices, which are structurally connected with the substantia nigra pars reticulata.
